# Deep Learning Algorithms with LIME and Similarity Distance Analysis on COVID-19 Chest X-ray Dataset

**DOI:** 10.3390/ijerph20054330

**Published:** 2023-02-28

**Authors:** Kuan-Yung Chen, Hsi-Chieh Lee, Tsung-Chieh Lin, Chih-Ying Lee, Zih-Ping Ho

**Affiliations:** 1 Department of Radiology, Chang Bing Show Chwan Memorial Hospital, Changhua 505, Taiwan; 2 Department of Computer Science and Information Engineering, National Quemoy University, Kinmen County 892, Taiwan; 3 College of Bioresources and Agriculture, National Taiwan University, Taipei 106, Taiwan; 4 Department of Business Administration, Chihlee University of Technology, New Taipei City 220, Taiwan

**Keywords:** COVID-19, LIME (Local Interpretable Model-agnostic Explanations), feature space, machine learning, outlier, PCA, similarity distance, U-Net segmentation

## Abstract

In the last few years, many types of research have been conducted on the most harmful pandemic, COVID-19. Machine learning approaches have been applied to investigate chest X-rays of COVID-19 patients in many respects. This study focuses on the deep learning algorithm from the standpoint of feature space and similarity analysis. Firstly, we utilized Local Interpretable Model-agnostic Explanations (LIME) to justify the necessity of the region of interest (ROI) process and further prepared ROI via U-Net segmentation that masked out non-lung areas of images to prevent the classifier from being distracted by irrelevant features. The experimental results were promising, with detection performance reaching an overall accuracy of 95.5%, a sensitivity of 98.4%, a precision of 94.7%, and an F1 score of 96.5% on the COVID-19 category. Secondly, we applied similarity analysis to identify outliers and further provided an objective confidence reference specific to the similarity distance to centers or boundaries of clusters while inferring. Finally, the experimental results suggested putting more effort into enhancing the low-accuracy subspace locally, which is identified by the similarity distance to the centers. The experimental results were promising, and based on those perspectives, our approach could be more flexible to deploy dedicated classifiers specific to different subspaces instead of one rigid end-to-end black box model for all feature space.

## 1. Introduction

Since December 2019, coronavirus pneumonia has spread from Wuhan, Mainland China. We call this COVID-19, and the International Committee on Taxonomy of Viruses (ICTV) defines this virus as SARS-CoV-2 (Acute Respiratory Syndrome Coronavirus 2). As of 21 December 2022, the number of infections worldwide is approximately 650 million, and the death toll is approximately 6.7 million [1]. All aspects of global politics, the economy, and society have been deeply affected. This research expects that machine learning can make auxiliary contributions to related detection or pathological development research, especially when professional medical personnel and testing resources are insufficient.

In this study, we used LIME to illustrate that classifiers are easily distracted by areas out of the lungs if without ROI preprocessing. Hence, we further trained the U-Net segmentation model to perform better ROI preprocessing. Compared with digital image processing, U-Net could significantly improve the effectiveness and efficiency of segmentation jobs.

We aim to develop a high-accuracy detection model for positive cases of COVID-19 patients. The novelties in this study are: (1) We trained an effectively deep-learning segmentation model in the pre-processing phase to mask out areas of the lung area that may distract the model’s learning. (2) We focused on similarity analysis to identify outliers and low accuracy subspace and further enhance the detection performance of the deep learning detection model locally. None of the researchers in the next section studied the relationship between cluster distribution and detection accuracy in feature space in terms of similarity distance.

## 2. Related Work

Since the outbreak of coronavirus pneumonia, the epidemic prevention measures adopted by various countries have not only restricted or controlled social activities but also adopted measures such as locking down the city or controlling daily access. At present, the related detection techniques [2] are divided into three types: reverse transcriptase polymerase chain reaction (PCR) nucleic acid detection, antigen detection, and serum IgM/IgG antibody detection. It can be seen that in the management of epidemic prevention and diversion, nucleic acid testing and rapid antigen screening are the main methods.

In terms of radiological detection, computed tomography (CT) and X-ray tests have also been studied in the detection and research of patients with coronavirus pneumonia. The paper [3] studied the correlation between CT and PCR detection to understand whether radiological detection has sufficient accuracy to replace or complement each other. X-ray is a cheaper and easier method than CT, and it is used more frequently and easily by various hospitals and patients.

The paper [4] summarized and compared machine learning algorithms used in medical imaging, covering traditional machine learning and deep learning. As to the most critical feature extraction work, traditional machine learning depends largely on the feature engineering skills of skilled operators.

In the development history of computer vision, deep learning has been greatly improved by the computing power of computers in the past decade, which has allowed it to show excellent learning results and be used in various fields. Among them, it has also become an important auxiliary tool of medical diagnosis [5]. The deep convolutional neural network is significantly better than other types of machine learning methods in terms of image feature extraction. The feasibility of using pre-trained weights for deep learning in medical images as transfer learning has also been verified in this study [6], and its application in pneumonia-related applications has also been verified [7]. As more and more open public datasets are available, the research speed in this part is even faster globally.

During the past and the coronavirus epidemic, the feasibility of deep convolutional neural network (CNN) medical contribution has been continuously studied. Gozes et al. [8] studied an off-the-shelf 3D CT analysis system to detect nodules and small opacities with a 2D complementary deep learning network to detect diffuse opacities, and further defined a score as a reference of illness progression over time. Chowdhury et al. [9] presented the comparison of different deep CNNs with transfer learning for COVID-19 detection. Hemdan et al. [10] used X-ray images to compare and select suitable COVIDX-Net models from seven different well-known models. However, due to the very limited dataset, it was studied only for training and validation, and the generalization is still challenging. Sachin et al. [11] used the DarkNet-based network, which was designed for only looking at one (YOLO) object detection as the backbone of the proposed DarkCovidNet classification. Wang et al. [12] proposed COVID-Net, which is a state-of-the-art customized CNN model on chest X-ray images and uses the automatic learning function to search for the optimization of the model structure. Additionally, during the epidemic, it also contributed a lot to the establishment of the public access dataset, COVIDx. Tang et al. [13] proposed the ensemble-based EDL-COVID network with several different snapshots over training epochs on a single COVID-Net model structure to boost performance. They used a more aggressive learning rate to diversify the detection behaviors of different snapshots.

Ensemble learning can aggregate different classifiers and can be used to integrate complex deep-learning models [14,15]. The main principle and contribution of its technology are to integrate grouped diverse models, grouped models with the same data set but in different views, or grouped models trained by different data subsets to achieve multi-pattern output aggregation.

Lin et al. [16] used the mask to preprocess ROI via traditional digital image processing. 

The decision-making process of most machine learning methods remains a black box. It is critical to understand or justify whether the detection behavior is reasonable, interpretable, and trustable. Generally, SHapley Additive exPlanations (SHAP) [17], Gradient-Weighted Class Activation Mapping (Grad-CAM) [18], and Local Interpretable Model-agnostic Explanations (LIME) [19,20] are well-known tools for interpretability analysis in different applications. SHAP analysis is an extension based on Shapley values and a game-theoretic method to calculate the average of all marginal contributions in all the coalitional combinations. However, for image analysis and deep learning architecture, it would take too many computation resources. Grad-CAM can obtain a good heatmap but it is not a model-specific method and is not flexible to frequent model adjustments. In contrast, LIME could obtain advantages of computation and flexibility if we do not need a fine-gram heatmap. 

LIME is a local surrogate model which is trained to approximate the detections of another complex underlying model. While training, LIME will first give variations of input data by perturbations and collect corresponding detections, then train an interpretable model with those data and weights of their proximity. 

## 3. Materials and Methods

**Figure 1:**
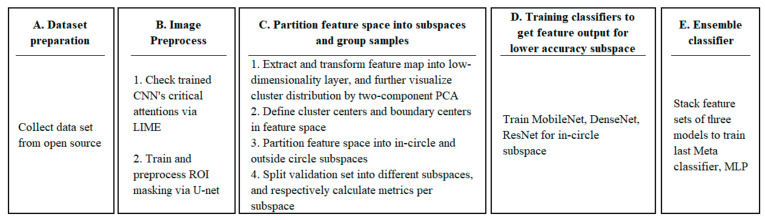
Training steps of this research.

The systematic architecture and operation process of this study are shown in Figure 1. For the training process, we started with step A collecting datasets from the open source, then followed with step B to initially justify the necessity of the ROI process. U-Net [21] was introduced to produce an ROI mask for each image. In step C, we trained a CNN model to produce feature vectors of images and partitioned feature space into low- and high-accuracy subspaces according to cluster statistics. Step D trained classifiers for the testing set falling in low-accuracy subspace and enhanced performance by stacking ensemble [22] in step E.

**Figure 2:**
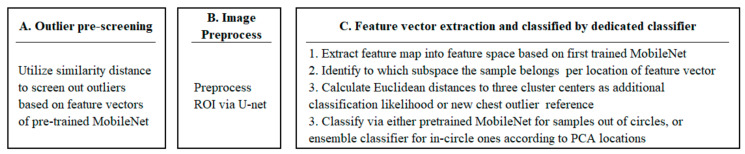
Inferring steps of this research.

Regarding inferring in Figure 2, we performed initial screening for outliers with feature distance to boundary centers [23], then preprocessed images with a trained U-Net model, and finally classified them with dedicated classifiers according to the location of their feature vectors.

### 3.1. Dataset Description

**Figure 3:**
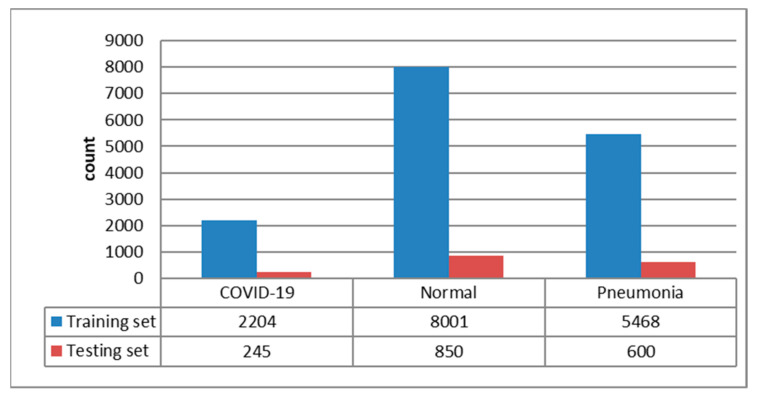
Sample distribution of this research. We roughly split 10% of the original data into the testing set.

We collected the dataset from the open access benchmark COVIDx8 dataset constructed in May 2022, which contains 17,368 PA Chest X-rays, of which 2449 belong to COVID-19, shown in Figure 3. The detailed metadata was not collected and analyzed in this open source. This dataset is modified and kept updated from five publicly available repositories [24,25,26,27,28]. The repository built by Cohen et al. [24] collected COVID-19 images and other viral and bacterial types of pneumonia images. COVID-Net Team built [25,26] repositories with about 300 COVID-19 images. Qatar University, the University of Dhaka, and researchers from Pakistan and Malaysia cooperated to build this repository [27]. RNSA launched a Kaggle competition with this dataset [28]. Please refer to the link for more COVIDx8-version dataset creating details (https://github.com/lindawangg/COVID-Net/blob/master/docs/COVIDx.md, accessed on 29 October 2022). 

The dataset has a significant sample imbalance among categories, particularly COVID-19 with a smaller quantity. During training, we adjusted class weights for loss function to balance the categories’ contribution levels. We roughly split 10% into the testing set.

### 3.2. Methods

Our experiments were conducted in the Intelligent Computing laboratory of National Quemoy University. We used Python-based TensorFlow framework for neural network layers, OpenCV for image processing, and sci-kit-learn for machine learning algorithms. Our computation machine is equipped with two GeForce GTX 1080 Ti GPUs. 

#### 3.2.1. Model Training Phase

- Step A: Dataset preparation;

The dataset was collected as previously mentioned from open sources.

- Step B: Image preprocessing;

For justifying the masking contribution of ROI, in the beginning, we trained MobileNetV1 [29] with original images without the ROI process and further applied LIME to visualize paid attention to images. In the process of LIME to each concerned image, we used the watershed algorithm to produce 50 pieces of small segmentations per image, then LIME randomly generated 150 perturbations by super-pixels on and off. By feeding those 150 perturbations into trained MobileNetV1, we could obtain the predicted probability for each class. A linear model is used to fit those 150 images as a model-agnostic model. Four top features were selected to show super-pixel images for our visual check.

U-Net is a semantic segmentation approach consisting of down-sampling and up-sampling phases by stacking convolutional layers. The novelty of this architecture is to concatenate higher resolution feature maps from down-sampling stages with up-sampled features by skipping connections to obtain richer information that is likely missing in the main path. Compared with digital image processing, U-Net could significantly improve the effectiveness and efficiency of segmentation jobs.

For the ROI masking process, we used 300 images selected from the same retrieved COVIDx8 dataset with our annotations to train the U-Net model to produce a segmentation mask and further used it to generate ROI masks for all the rest images.

Even though we have thousands of image data, the resources for annotation work are still limited. While U-Net is capable of performing excellent segmentations, detailed and accurate annotations still require the help of professional staff. Therefore, in this study, we targeted preliminarily and smoothly masked out areas outside lung edges.

- Step C: Partition feature space into subspaces and group samples;

We trained MobileNetV1 with 224 × 224 ROI images, ImageNet pre-trained weights of feature extraction blocks, additional head layers for three-category classification, 0.0001 learning rate, and categorical cross entropy as loss function, then used the new-trained model to produce the feature vectors of all training data from a low-dimensionality layer with 20 perceptrons just after the last convolutional layer. From the distribution of clusters in feature space, we defined centers of category clusters and boundaries. Cluster centers were identified by K-means and boundary centers were set in the middle positions between cluster centers. Meanwhile, we also introduced Principal Component Analysis (PCA) [30] to visualize the distribution of clusters in two-component dimensions. Based on centers and L2 Euclidean distance to cluster centers for each sample, we can obtain much more valuable information as likelihood or new chest outlier reference in addition to simple category classification answers.

We further partitioned feature space into two sub-spaces as in-circle and outside-circle areas, according to Euclidean distance to boundary centers. Through the feature extraction of trained MobileNetV1, samples falling in in-circle boundary areas would basically have much lower accuracy. Because the training set would be well clustered and likely over-fitted, it is better to take the detection accuracy of the testing sample as an objective accuracy reference.

- Step D: Training classifiers to obtain feature output for lower accuracy subspace;

For enhancing the detection accuracy of in-circle subspace, we tried to use stacking ensemble and data augmentation. Therefore, we selected and trained images sized 224 × 224 with well-known MobileNetV1, ResNet50V2 [31,32], and DenseNet201 [33], respectively, to enrich diversity, which is required by the ensemble approach. 

MobileNet is a lightweight model designed for edge devices by Google in 2017. By splitting the general 2D convolution mode into an independent 3 × 3 depth-wise convolution for each channel, combined with 1 × 1 point-wise convolution, a similar performance can be achieved, but the amount of calculation is greatly reduced. 

ResNet uses the residual block architecture to enable neural networks to surpass human capabilities in visual recognition for the first time, and also won the championship of ImageNet2015 and COCO competition and the best paper of CVPR2016. The original design purpose is to use the residual calculation to pass the input to the output, reducing the problem of gradient disappearance or explosion in deep structure training. ResNet50 uses 50 convolutional layers for feature extraction. V2 is the version of operation sequence adjustment on residual units based on V1.

DenseNet uses a skip connection mechanism similar to ResNet. The difference is that DenseNet uses all the previous layers to concatenate to recombine features, and these dense jumper connections are why we call it “Dense”. DenseNet201 has 201 trainable layers.

In data augmentation, we used the skills of width shift, height shift, and horizontal flip. At the end of this step, we can obtain three sets of feature vectors in the middle layer we are interested in for the next step.

- Step E: Ensemble classifier.

Here, we concatenated the above three classifiers’ feature vectors, probability, or logits output and further fed it as the meta-classifier’s training input. Multi-layer perceptron (MLP) was used as the last meta-classifier.

In our study, we mainly evaluated three metrics. Accuracy = (TP + TN)/(TP + TN + FP + FN). Precision = TP/(TP + FP). Recall = TP/(TP + FN). Where TP, TN, FP, and FN stand for True Positive, True Negative, False Positive, and False Negative, respectively.

#### 3.2.2. Inferring Phase

- Step A: Outlier pre-screening;

Similarity distance is a potential tool to identify outliers that completely do not belong to any category in case any wrong input operation is there. The CNN classification model is incapable of screening out for ridiculous outliers. We implemented this pre-screening before feeding images into classifiers. Twelve outlier images were selected from Google to observe the effectiveness and performance of this gating. The Imagenet-pre-trained MobileNetV1 is leveraged here.

- Step B: Image Preprocess;

As in the training phase, we need to perform ROI masking for testing data.

- Step C: Feature vector extraction and classification by a dedicated classifier.

We extracted feature vectors of images and classified them with different classifiers customized per subspace of feature vector location based on the first trained MobileNetV1. Moreover, we could calculate Euclidean distances to three cluster centers as additional information on classification likelihood or new chest outlier reference.

## 4. Results

### 4.1. Model Training

**Figure 4:**
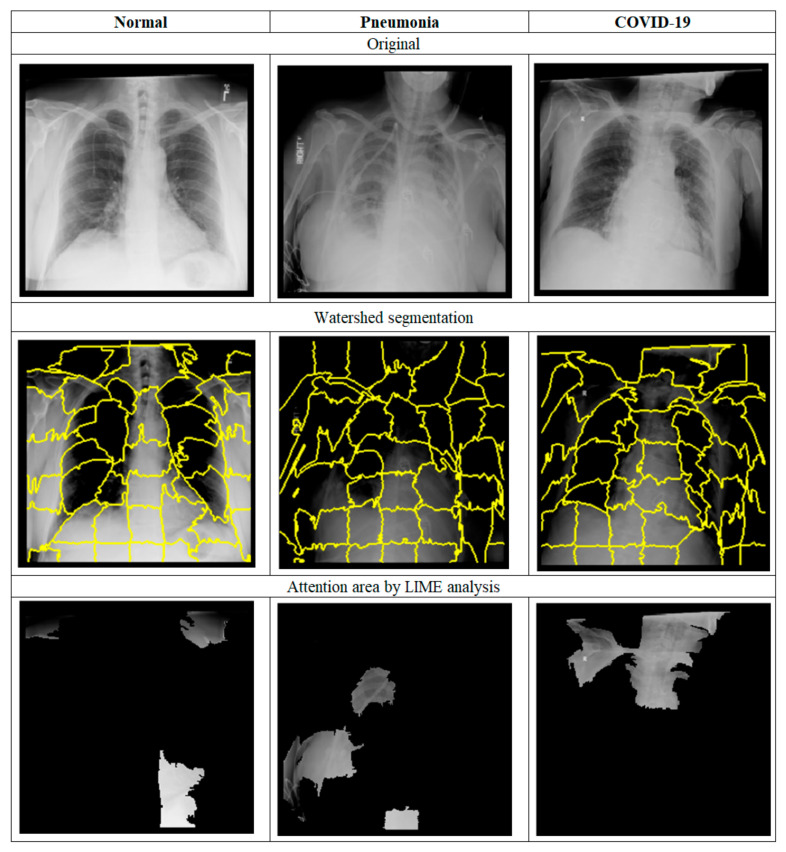
Images for the original Chest X-ray, watershed segmentation, and the results after the LIME analysis.

**Figure 5:**
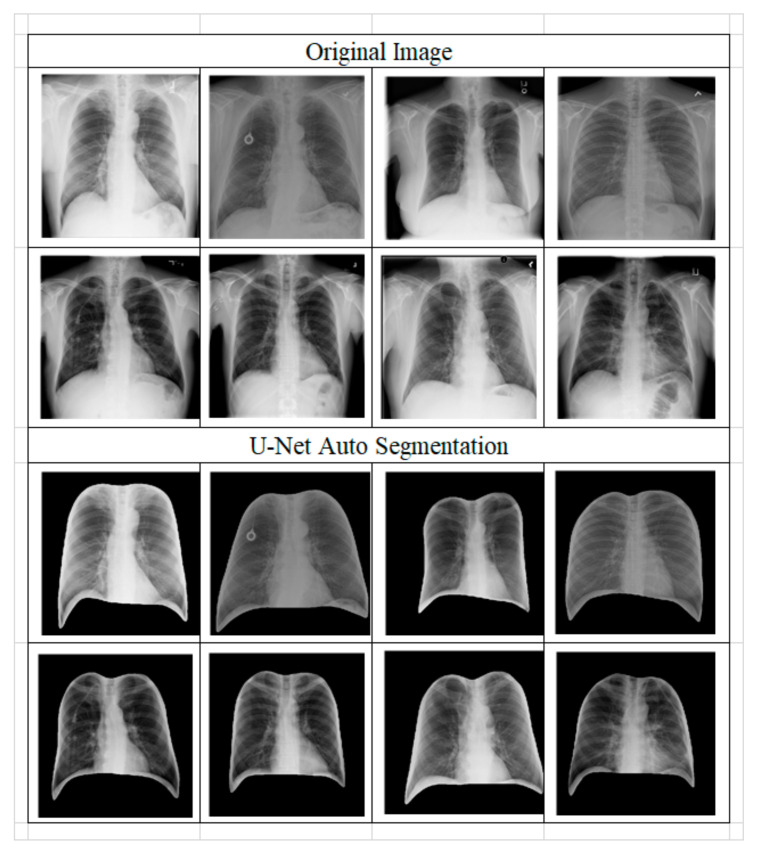
Images for the result of ROI preprocess by U-Net.

From Figure 4, LIME analysis shows that the classification model sometimes made a critical judgment based on an area out of the lungs that are not in compliance with our intention, even though the classifier reached high accuracy. This situation will cause serious generalization issues. We obtained the conclusion about the necessary ROI to preprocess from LIME analysis and proceeded with U-Net. The effect is in Figure 5, which shows an excellent masking result smoothly along with the lungs’ edge.

**Figure 6:**
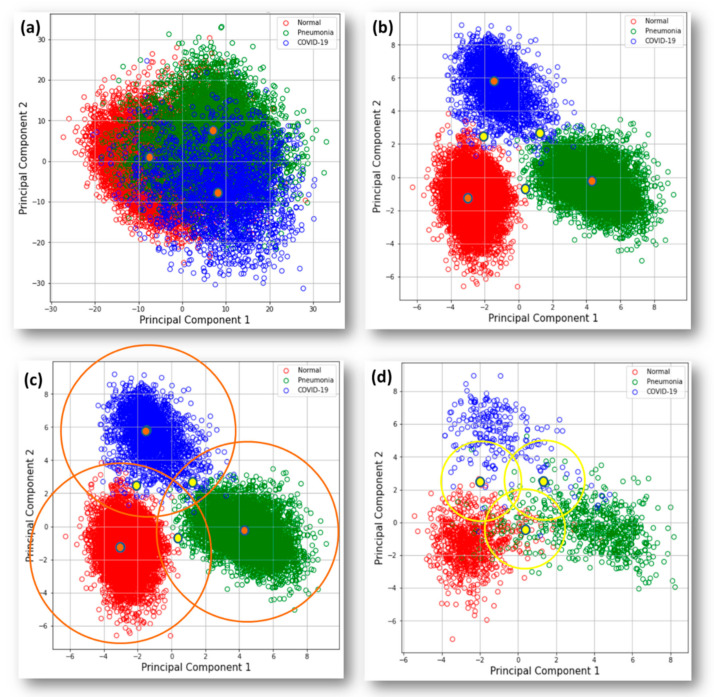
PCA visualization of the training set clusters in two-component: (**a**) Down-sampling distribution based on extracted 1000 vectors with MobilNetV1 pre-trained by Imagenet. (**b**) Status based on trained MobileNetV1 feature extraction block. It also shows cluster centers in orange and boundary centers in yellow. (**c**) Orange circles are the outlier boundaries specific to different categories. (**d**) Yellow circles are boundary sketches to separate both high and low-accuracy subspaces.

In step C, although we handled those data with high dimensionality, meanwhile, we also used PCA to drop those vectors into two components for visual observation. In Figure 6b, we can observe clusters of the training set well separated into three categories, defined cluster centers in orange and boundary centers in yellow. In Figure 6c, orange circles represent new chest outlier boundaries. In Figure 6d, yellow circles are the boundaries between high- and low-accuracy subspaces.

### 4.2. Inferring

**Figure 7:**
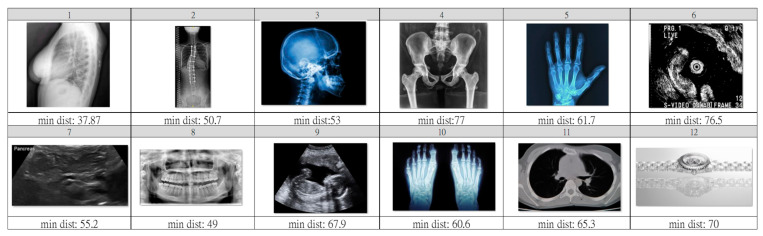
Outlier set and minimum L2 distance to three cluster centers. The number one image, lateral view X-ray, has the shortest similarity distance.

For effective observation of outlier pre-screening in step A, we selected twelve images that are quite different from the chest X-ray. From our experiment, the ImageNet pre-trained weight of MobileNetV1 for convolutional layers can work very well with the 1000 feature vectors we directly leveraged. Figure 6a shows PCA down-sampling for the features extracted from the pre-trained model. Compared with Figure 6b, we can clearly observe poor clustering performance on our three chest categories. Namely, it is necessary to train the model for our use case in the next step. Here, we set L2 distance 45 as the threshold that could screen out less than 1 percent for both training and testing sets, but that reached a good screening out rate to the outlier set. In Figure 7, we list the minimum distance to cluster centers and images of outliers. Among these, the lateral-view chest X-ray of number one is the only one below the threshold. It is somehow reasonable due to its lung features that have similar features to our dataset.

**Figure 8:**
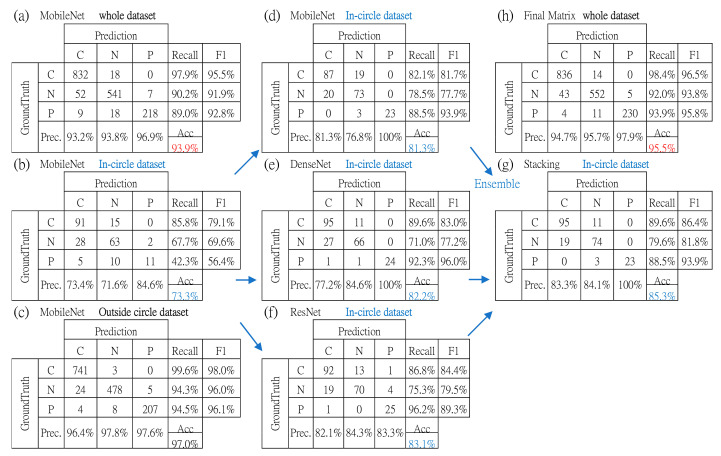
All relevant confusion matrices: (**a**–**c**) are for status before partition enhancement, (**d**–**f**) are the improvement of (**b**) with data augmentation on three models, (**g**) is the result of the ensemble, and (**h**) is the final result for all datasets. Dataset here means testing dataset.

In classification step C, Figure 8a–c respectively shows the confusion matrix of the testing set under conditions of all in-circle and outside-circle space. The in-circle matrix shows the worst result for all metrics. While inferring application, samples in this in-circle area can be highlighted as low-confidence detection in addition to the simple result of the predicted class. With it, the user can plan future checks per confidence level.

Figure 8d–f shows three individual CNN, MobileNetV1, DenseNet201, and ResNet50V2 with data augmentation, and synergy of stacking ensemble in Figure 8g. Figure 8h is the combination of Figure 8b,g as the final performance for all testing samples.

For the accuracy of the in-circle area, improvement is from 73.3% to 85.3%, whereas the accuracy of the whole area is from 93.9% to 95.5%. Table 1 shows the contribution of the stacking ensemble in step C.

## 5. Discussion

DarkCovidNet [11] could achieve 87.02% accuracy for the three-class classification. This main algorithm is DarkNet, which is designed for YOLO. COVID-Net [12] is a tailor-made residual deep learning architecture with optimization of generative synthesis strategy for COVID-19 detection that achieved an accuracy of 93.3%. EDL-COVID [13] is a snapshot ensemble deep learning model based on COVID-Net that reached an accuracy of 95%. We listed our results with those state-of-the-art methods in Table 2.

From previous related work [7,8,9,10,11,12,13], many types of research on this COVID-19 chest X-ray topic did not focus on the ROI preprocessing and possibly introduced the risk of utilizing features out of the lung area while implementing classification with CNN. Normally, from our study, we observed that 2% accuracy will be lost if introducing ROI preprocessing, which means models trained without an ROI dataset would make a judgment based on the irrelevant area on images and likely lead to generalization issues in future applications. In our study, LIME analysis shows the same comments as the research with Grad-CAM analysis [16]. It justified our deep learning ROI approach, U-Net segmentation, as a necessary method in this application. In the future, LIME is also an alternative to Grad-CAM if we aim to target lesion areas inside ROI in the macro view.

We deeply take advantage of feature space distribution to analyze outliers with a big difference, visualize clusters and cluster overlap, and partition the whole feature space into different levels of detection confidence based on the testing set. For outlier detection, we simply leveraged the pre-trained model to obtain a high screening out rate on our outlier set and with low impact, less than 1%, to original chest X-ray images. Furthermore, in another feature space of the re-trained model, it could be leveraged to identify new chest illness classes other than trained classes on chest X-rays based on the proper distance threshold to cluster centers. From our approach, similarity distance to boundary centers could effectively distinguish different detection levels based on a testing set as a meaningful confidence reference. In the future, we can refine the current two-level partition into finer levels for better confidence resolution. 

Moreover, to each subspace, we can treat them with different intentions or biases without impacting other trained models as partial finetune. In this study, we aimed to enhance the accuracy of low-accuracy subspace with data augmentation and ensemble, which resulted in an improvement from 73.3% to 85.3% locally. In the future, a better way for the enhancement of low accuracy subspace is suggested to deeply analyze the difference between feature vectors of the training set and testing set falling into this area and further improve the combination or image quality of the training set to enhance specific subspace.

This study has limitations from the collected dataset. Firstly, image quality and lesions of chest X-rays were not well-checked or annotated by professional medical staff. This will lead to a difficult review or discussion if we want to improve the model, especially for critical samples around cluster boundaries. Secondly, regarding the limited sample size, even though we utilized sample weight to balance and shift the model focus on the most concerned category while training, the model still possibly could not learn important discriminative features from a limited sample size if they are not there. Thirdly, COVID-19 has many variants that would possibly damage a patient’s lungs in different ways in different stages. Fourthly, the COVIDx dataset is kept updated without a detailed metadata log, so it is difficult to combine more useful meta information for better learning. However, even with mentioned limits, we still think this feature-space-based similarity analysis could provide much more reference information than simple classification results and provide flexibility to fine-tune partial functions with our strategic bias. 

## 6. Conclusions

In this study, we utilized cluster distribution in feature space to identify outliers and further split feature space into high- and low-accuracy subspaces based on the testing dataset. In this way, for predicting future samples, more than simply predicted category results, we can obtain more objective detection confidence reference according to similarity distance to centers. Furthermore, we can flexibly deploy different classifiers to different subspaces according to different strategies or biases without interfering with other subspaces, instead of one rigid end-to-end model approach for all. In this case, we tried to enhance classification performance for low-accuracy subspace.

We also used LIME to justify the necessity of the ROI preprocessing that is based on the deep learning U-Net generator. Approaches without an ROI process may include generalization risk due to the chance of using features out of the lung area.

## Figures and Tables

**Table 1:** Performance comparison of individual CNN to the ensemble.

In-Circle Testing Dataset	Image Size	Overall Accuracy	Avg. Sensitivity of All	Avg. Precision of All	Avg. F-Score of All	Sensitivity of COVID-19
Individual CNN Stage						
MobileNet	224 × 224	81.3%	83.0%	86.1%	84.4%	82.1%
DenseNet	224 × 224	82.2%	84.3%	87.3%	85.4%	89.6%
ResNet	224 × 224	83.1%	86.1%	83.3%	84.4%	86.8%
Ensemble stage						
Stacking with MLP	NA	85.3%	85.9%	89.1%	87.3%	89.6%

**Table 2:** Detection performance comparison among three state-of-the-art methods, our based-line MobileNet50V1, and the proposed model.

Method	Accuracy	Sensitivity	Precision
DarkCovidNet	88.6%	89.0%	94.6%
COVID-Net	93.3%	91.0%	98.9%
EDL-COVID	95.0%	96.0%	94.1%
MobileNet50V1	93.9%	97.9%	93.2%
Proposed-model	95.9%	98.4%	94.7%

## Data Availability

The data presented in this study are available on request from the corresponding author.
